# Effects of dexamethasone and meloxicam on *Borrelia burgdorferi*-induced inflammation in glial and neuronal cells of the central nervous system

**DOI:** 10.1186/s12974-017-0806-9

**Published:** 2017-02-02

**Authors:** Geeta Ramesh, Alejandra N. Martinez, Dale S. Martin, Mario T. Philipp

**Affiliations:** 1 Division of Bacteriology and Parasitology, Tulane National Primate Research Center, Covington, LA USA; 2 Department of Microbiology and Immunology, Tulane University Medical School, New Orleans, LA USA

**Keywords:** Lyme neuroborreliosis, *Borrelia burgdorferi*, Inflammation, Apoptosis, Dexamethasone, Meloxicam

## Abstract

**Background:**

Lyme neuroborreliosis (LNB), caused by the spirochete *Borrelia burgdorferi* (Bb), affects both the central and peripheral nervous systems. Previously, we reported that in a model of acute LNB in rhesus monkeys, treatment with the anti-inflammatory drug dexamethasone significantly reduced both pleocytosis and levels of cerebrospinal fluid (CSF) immune mediators that were induced by Bb. Dexamethasone also inhibited the formation of inflammatory, neurodegenerative, and demyelinating lesions in the brain and spinal cord of these animals. In contrast, these signs were evident in the infected animals that were left untreated or in those that were treated with meloxicam, a non-steroidal anti-inflammatory drug.

**Methods:**

To address the differential anti-inflammatory effects of dexamethasone and meloxicam in the central nervous system (CNS), we evaluated the potential of these drugs to alter the levels of Bb-induced inflammatory mediators in culture supernatants of rhesus frontal cortex (FC) explants, primary rhesus astrocytes and microglia, and human oligodendrocytes. We also ascertained the potential of dexamethasone to modulate Bb-induced apoptosis in rhesus FC explants. As meloxicam is a known COX-2 inhibitor, we evaluated whether meloxicam altered the levels of COX-2 as induced by live Bb in cell lysates of primary rhesus astrocytes and microglia.

**Results:**

Dexamethasone but not meloxicam significantly reduced the levels of several Bb-induced immune mediators in culture supernatants of FC explants, astrocytes, microglia, and oligodendrocytes. Dexamethasone also had a protective effect on Bb-induced neuronal and oligodendrocyte apoptosis in rhesus FC explants. Further, meloxicam significantly reduced the levels of Bb-induced COX-2 in microglia, while both Bb and meloxicam were unable to alter the constitutive levels of COX-2 in astrocytes.

**Conclusions:**

These data indicate that dexamethasone and meloxicam have differential anti-inflammatory effects on Bb-induced inflammation in glial and neuronal cells of the CNS and help explain the in vivo findings of significantly reduced inflammatory mediators in the CSF and lack of inflammatory neurodegenerative lesions in the brain and spinal cord of Bb-infected animals that were treated with dexamethasone but not meloxicam. Signaling cascades altered by dexamethasone could serve as possible therapeutic targets for limiting CNS inflammation and tissue damage in LNB.

## Background

Lyme disease is caused by infection with the spirochete *Borrelia burgdorferi* (Bb) [1]. The nervous system involvement in Lyme disease, called Lyme neuroborreliosis (LNB), may affect both the central and peripheral nervous systems in about 15% of Lyme disease patients. Symptoms of acute LNB include painful meningoradiculitis with inflammation of dorsal nerve roots and lancinating, radicular pain (Bannwarth’s syndrome), lymphocytic meningitis, and various forms of cranial or peripheral neuritis [2].

The rhesus macaque is the most accurate model of human LNB [3–6]. Previously, we reported that leptomeningitis and radiculitis that manifest in monkeys with acute LNB are concomitant with the inflammatory meditator response elicited by Bb [6]. Importantly, lymphocyte and plasma cell infiltration in the leptomeninges and perivascular infiltrates of immune cells adjacent to white matter lesions in the brain and transverse myelitis lesions in the spinal cord have been documented in pathological examinations of lesions from cases of human LNB [2, 7, 8].

We hypothesized that Bb induces the production of inflammatory mediators in glial and neuronal cells and that this response has a role in potentiating glial and neuronal apoptosis. We recently explored if inflammation had a causal role in mediating the pathogenesis of LNB by evaluating the inflammatory changes in rhesus macaques infected with Bb that were left untreated or were given either the anti-inflammatory drug dexamethasone, a steroid that inhibits the expression of several immune mediators [9], or meloxicam, a non-steroidal anti-inflammatory drug that inhibits cyclooxygenase-2 (COX-2) [10]. Dexamethasone treatment significantly reduced the levels of several cytokines and chemokines, and pleocytosis in the CSF, and prevented inflammatory and/or neurodegenerative and demyelinating lesions in the central and peripheral nervous systems [11]. Conversely, infected animals that were treated with meloxicam showed similar levels of immune mediators in the CSF and displayed similar lesions in the CNS and PNS to those seen in infected animals that were left untreated. Also, the effects of these drugs in neuronal cultures of dorsal root ganglia and of myelinating cells of the PNS infected with Bb showed that dexamethasone but not meloxicam significantly reduces the levels of apoptosis and those of several cytokines and chemokines [12].

In this study, we evaluate the effects of these drugs on Bb-induced inflammation in glial and neuronal cells of the CNS. Results show that dexamethasone but not meloxicam significantly reduces the levels of several cytokines and chemokines as induced by live Bb in rhesus astrocytes, microglia, and FC explants, in addition to human oligodendrocytes. Likewise, dexamethasone showed a protective effect on cell death, as both neurons and oligodendrocytes evinced reduced apoptosis in rhesus FC explants. Further, meloxicam was able to significantly reduce the levels of Bb-induced COX-2 in rhesus microglia, while it did not alter the constitutive levels of COX-2 in rhesus astrocytes. These data indicate that as with PNS cells [13], dexamethasone and meloxicam have differential anti-inflammatory effects on Bb-induced inflammation in glial and neuronal cells of the CNS.

## Methods

### Growth and preparation of live spirochetes

*B. burgdorferi* B31 clone 5A19 spirochetes, passage 1–3, were grown in Barbour-Stoenner-Kelly (BSK) medium, supplemented with 6% rabbit serum (Sigma, St. Louis, MO) and antibiotics (rifampicin at 45.4 μg/mL, phosphomycin at 193 μg/mL, and amphotericin at 0.25 μg/mL) to late logarithmic phase under microaerophilic conditions. Spirochetes were pelleted, washed using sterile phosphate-buffered saline (PBS), and resuspended in the working medium at the desired density, as previously described [12].

### Frontal cortex brain (FC) explant experiments: incubation of brain slices with *B. burgdorferi* (Bb), dexamethasone, and meloxicam for collection of culture supernatants for multiplex ELISA assays and apoptosis assays with tissue explants

Freshly harvested frontal cortex tissues were collected at necropsy from three rhesus macaques (*Macaca mulatta*) that were scheduled for euthanasia due to chronic idiopathic diarrhea or had undergone trauma. Animals were euthanized in accordance with the recommendations of the American Veterinary Medical Association’s Panel on Euthanasia. The frontal cortex was sliced into 2-mm sections, and each section was placed in separate wells of 12-well plates. Each well contained 2 mL of RPMI 1640 medium (BioWhittaker, Walkersville, MD) supplemented with 10% FBS, as previously described [14]. Tissue sections were exposed to medium alone or to medium with added Bb (1 × 10^7^ bacteria/mL) in the presence or absence of either 150 μM dexamethasone (Sigma) or 100 μM meloxicam (Sigma). Controls with no spirochetes were also included. Prior to stimulation with live Bb, FC explant cultures were incubated with the respective concentrations of dexamethasone or meloxicam for 1 h in a humidified 5% CO_2_ incubator at 37 °C, after which they were washed and then incubated in fresh growth medium containing the appropriate concentrations of dexamethasone or meloxicam and live Bb for an additional 6 h at 37 °C. The above concentrations of dexamethasone and meloxicam were the highest concentrations that were found to be safe as evaluated by the trypan blue exclusion assay (Sigma) in previous studies with cultures of human Schwann cells and rhesus dorsal root ganglia cultures [13]. At the end of the incubation times, culture supernatants were collected for evaluation of inflammatory mediators. Culture supernatants were centrifuged at 4 °C at 2000 × *g* to remove any suspended bacteria, and the supernatant was aliquoted and stored at −70 °C until used, as previously reported [13]. Tissue explants in respective wells were fixed using 2% paraformaldehyde and cryopreserved for apoptosis assays.

### Primary cultures of rhesus astrocytes and microglia

Primary rhesus astrocyte and microglial cultures were established following previously described protocols [15]. Briefly, after the meninges and blood vessels from fresh frontal cortex tissue were removed, tissue was mechanically dissociated, treated with 0.25% trypsin, 0.38 g/mL EDTA (Invitrogen), and 0.1% DNase (Sigma-Aldrich), and incubated at 37 °C for 40 min with intermittent shaking. After incubation, the dissociated tissue was centrifuged for 10 min at 425 × *g* and cells filtered through a Nitex filter (20 μm) and resuspended in glial culture medium, which was composed of DMEM-F-12 with l-glutamine and HEPES buffer, 10% fetal bovine serum (HyClone), 0.5 ng/mL of granulocyte-macrophage colony-stimulating factor (Sigma-Aldrich), 100 U/mL penicillin, and 100 μg/mL streptomycin. After 14 to 21 days in culture at 37 °C, dislodged microglia were collected after vigorously tapping the flasks, and resuspended in glial culture medium.

To obtain purified astrocytes, glial cells were incubated for 90 min in 10 mM l-leucine methyl ester (LME) (Sigma-Aldrich). This concentration has been shown to be effective in ensuring maximal microglial lysis with minimal toxicity to astrocytes [15]. Thereafter, astrocytes were washed thoroughly and resuspended in glial culture medium. Purity of astrocytes and microglial cultures was assessed by staining with a specific microglial marker anti-IBA antibody at a concentration of 1:50 (Wako Chemicals, Richmond, VA) and was routinely of 99%. Astrocyte cultures were stained with astroglial marker anti-GFAP antibody at a dilution of 1:200 (Sigma) after permeabilizing fixed astrocyte cultures with a mixture of chilled ethanol-acetic acid mixture (2:1), for 5 min at −20 °C, for detection of intracellular phenotypic markers as previously described [16]. Confocal microscopy was performed using a Leica TCS SP8 confocal microscope (Leica Microsystems, Exton, PA).

### Human oligodendrocyte cultures

The human oligodendrocyte cell line MO3.13 obtained from CELLutions Biosystems Inc. (Burlington, Ontario, Canada) was revived and established following manufacturer’s instructions as previously described [17]. Differentiated oligodendrocytes were obtained after maintaining cultures in differentiation medium (DM), consisting of DMEM, P/S, and phorbol 12-myristate 13-acetate (Sigma), at a concentration of 100 nM and devoid of serum.

### Evaluating the anti-inflammatory potential of dexamethasone and meloxicam in rhesus astrocytes and microglia and human oligodendrocyte cultures stimulated with live Bb

Glial and Bb cultures were washed in their respective media, devoid of antibiotics. Controls with no spirochetes were also included. Prior to stimulation with live Bb, glial cell cultures were incubated with various concentrations of dexamethasone 5 μM, 15 μM and 150 μM (Sigma) or meloxicam 1 μM, 10 μM, 50 μM and 100 μM (Sigma) for 2 h at 37 °C, after which they were washed and then incubated in fresh growth medium containing the respective concentrations of dexamethasone or meloxicam and live Bb at a multiplicity of infection (MOI) of 10:1 at 37 °C. The effect of the carrier substance of dexamethasone (2-hydroxypropyl)-β-cyclodextrin), at the respective molar concentrations accompanying dexamethasone, was assessed by incubating rhesus astrocytes and microglial cultures in the presence and absence of Bb and carrier alone at 15, 45, and 450 μM, respectively, as previously described for oligodendrocyte cultures [16]. After 48 h, culture supernatants were collected and processed for evaluation of inflammatory mediators. The drug concentrations were chosen based on previous reports where dexamethasone (5–150 μM) had been shown to inhibit the production of CCL2 in mice microglia [18], and meloxicam (1–100 μM) had been shown to be effective in inhibiting COX-2 in vitro [19].

### Evaluation of immune mediators from culture supernatants

The concentrations of cytokines and chemokines present in the culture supernatants from rhesus astrocyte and microglial cultures and rhesus FC explant cultures were quantified using the MilliPlex MAP Non-Human Primate Cytokine Magnetic Bead Panel-Premixed 23 Plex, Cytokine-Chemokine Array kit (Millipore), following the manufacturer’s instructions. The concentrations of cytokines and chemokines present in the culture supernatants from human oligodendrocytes were quantified using the Human 14-plex Cytokine-Chemokine Array kit (Millipore), following the manufacturer’s instructions. The multiplex plate was read using a Bio-Plex 200 Suspension Array Luminex System (Bio-Rad, Hercules, CA, USA).

### Apoptosis by in situ TUNEL assay and confocal microscopy

Tissue sections from rhesus FC explants were incubated with anti-NeuN or anti-S-100 antibodies and then subjected to the TUNEL-ApopTagPlus fluorescein in situ apoptosis assay (Chemicon, Temecula, CA) as previously described [14]. The percentage of apoptotic neurons and the percentage of apoptotic oligodendrocytes in brain sections were evaluated by counting at least 500 cells, followed by the percentage of cells that showed co-localization of both the terminal deoxynucleotidyl transferase dUTP nick end labeling (TUNEL) signal and NeuN or S-100 expression, respectively. Apoptotic cells from ten fields were counted from each section for the various incubation conditions. All counts were made by viewing slides under a fixed magnification of ×63 (corresponding to an area of 0.05 mm^2^) using the Leica TCS SP8 confocal microscope. The identity of the oligodendrocytes that stained with the anti-S-100 antibody was confirmed by their morphology as previously described [14].

### COX-2 activity assay from culture lysates

Astrocytes and microglia cell lysates were prepared following the manufacturer’s instructions described in the COX Activity Assay Kit (Cayman Chemicals, Ann Arbor, MI). The Cayman’s COX activity assay kit measures the total peroxidase activity of COX in cell lysates and is assayed colorimetrically by monitoring the appearance of oxidized N, N, N′,N′-tetramethyl-*p*-phenylenediamine (TMPD) at 590 nm. The total COX-2 activity in the sample was calculated using the formula provided by the manufacturer.

### Statistical evaluation

The two-way ANOVA and Tukey’s multiple comparison test was used to evaluate the statistical significance between means of data sets, using Graphpad Prism software (Graph Pad Software Inc.) version 5.

## Results

### Effects of dexamethasone and meloxicam on the levels of cytokines and chemokines induced by Bb in rhesus frontal cortex explants

**Fig. 1:**
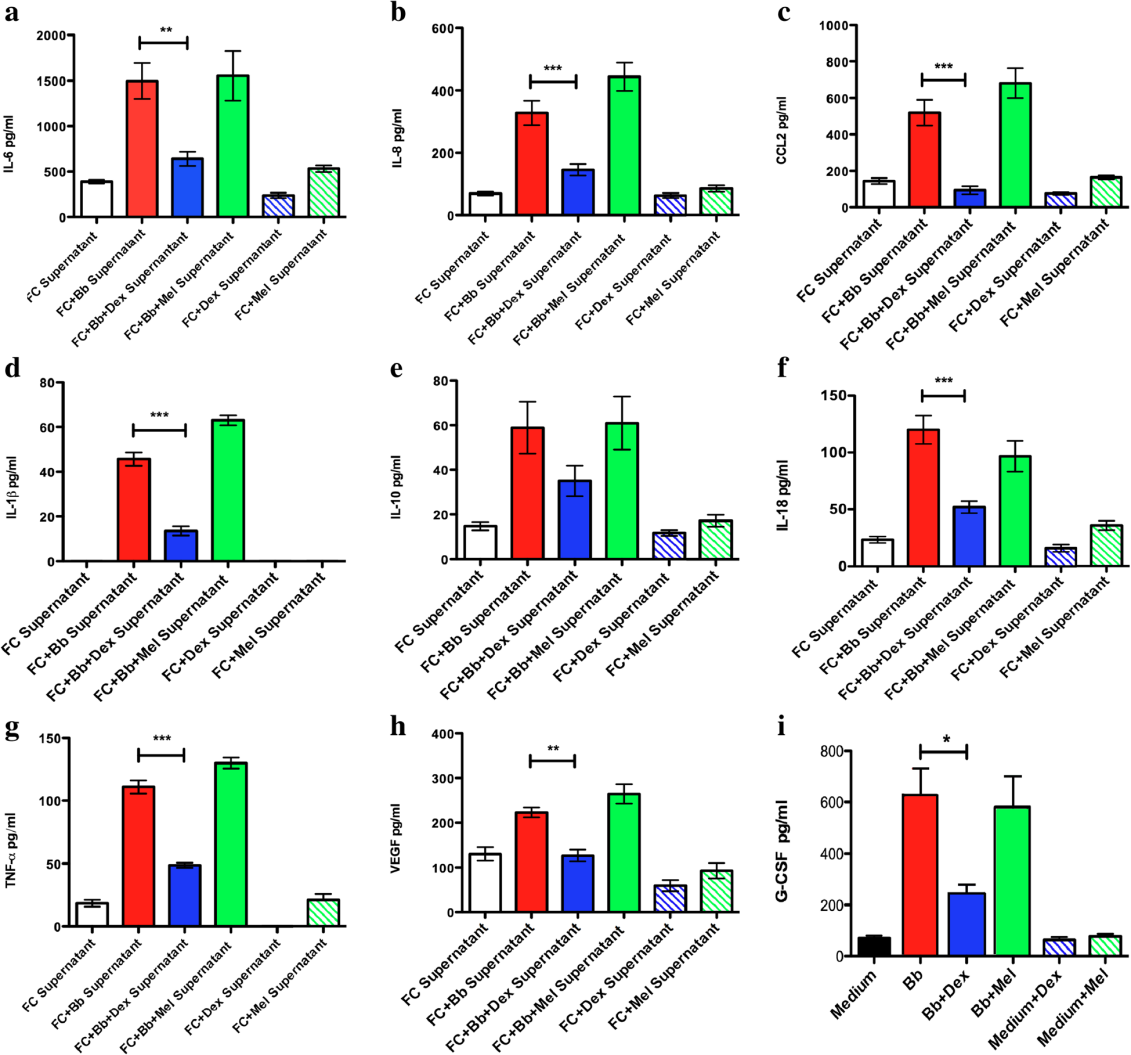
Dexamethasone but not meloxicam reduces the levels of Bb-induced cytokines and chemokines in frontal cortex explants after 6 h of incubation. The graphs represent the effect of the anti-inflammatory drugs on the levels of **a** IL-6, **b** IL-8, **c** CCL2, **d** IL-1β, **e** IL-10, **f** IL-18, **g** TNF-α, **h** VEGF, and **i** G-CSF. The two-way ANOVA and Tukey’s multiple comparison test were used to evaluate the statistical significance between means and SEM of triplicate data sets, *p < 0.05, **p < 0.01, ***p < 0.001

Culture supernatants from slices of the frontal cortex that were incubated with Bb and treated with dexamethasone resulted in significantly reduced levels of IL-6, IL-8, CCL2, IL-1β, IL-18, TNF-α, VEGF, and G-CSF, compared with slices that were incubated with Bb alone. In contrast, results from meloxicam treatment showed that this non-steroidal drug was unable to significantly reduce the levels of the pro-inflammatory cytokines and chemokines evaluated (Fig. 1a–i). The levels of IL-10, a cytokine known to counteract pro-inflammatory mediators, were also measured, and results showed that there were no significant differences after treatment with either one of the anti-inflammatory drugs (Fig. 1e). However, the level of IL-10 was lower after dexamethasone treatment than it was with Bb alone, albeit not significantly. The pattern of mediator response found in the tissue section supernatants was also present in the tissue lysates (data not shown).

### Effects of dexamethasone and meloxicam on the levels of CCL2 induced by Bb in cultures of rhesus astrocytes and microglia and human oligodendrocytes

**Fig. 2:**
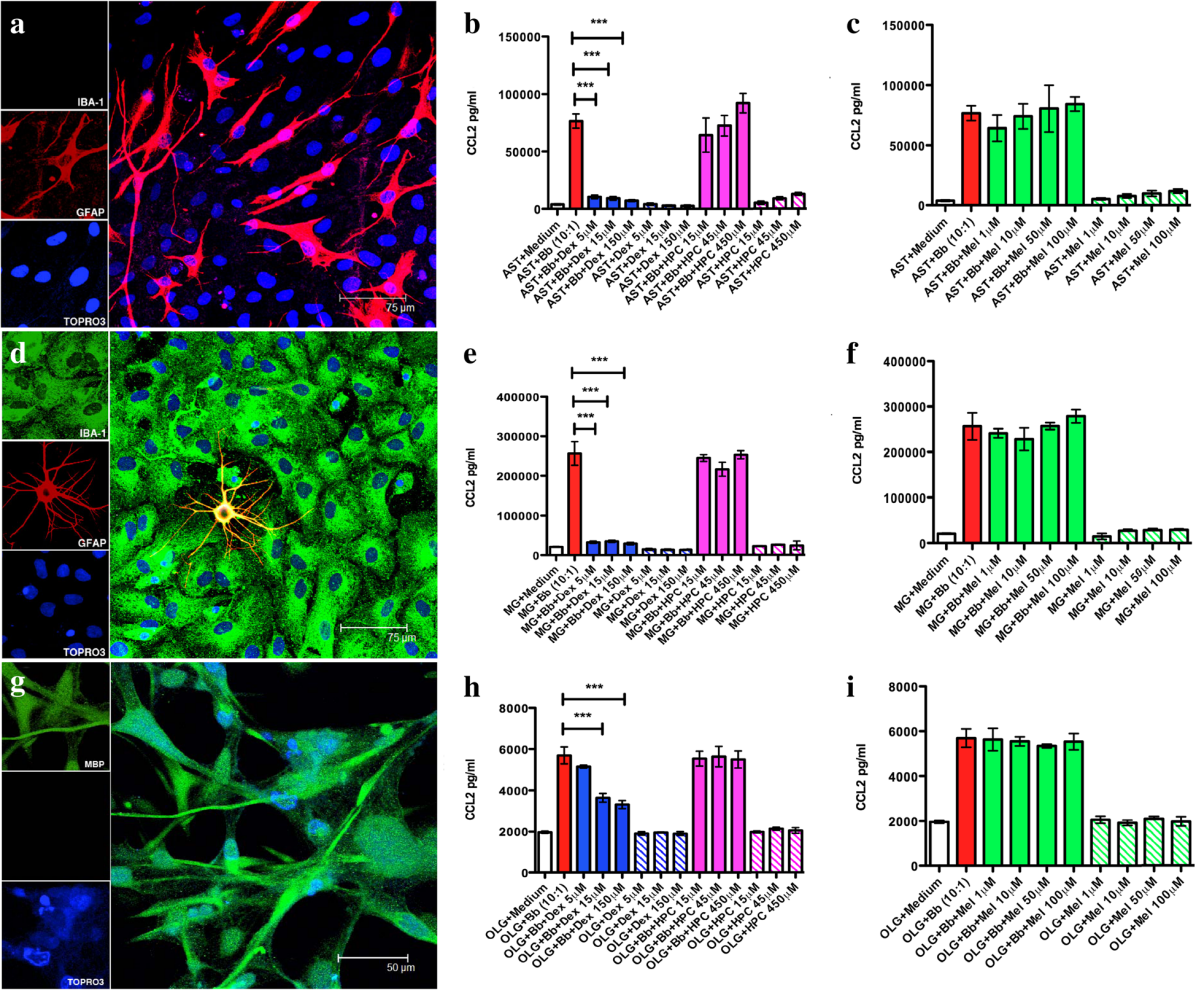
Dexamethasone but not meloxicam affects the levels of CCL2 induced by live Bb at a multiplicity of infection of 10:1 in in vitro cultures of rhesus astrocytes (**a**–**c**), rhesus microglia (**d**–**f**), and human oligodendrocytes (**g**–**i**) after 48 h of incubation. The effect of different concentrations of dexamethasone (**b**, **e**, **h**) and meloxicam (**c**, **f**, **i**) on the levels of CCL2 as induced by live Bb in different glial cells are represented in the graphs. Representative confocal micrographs showing cell morphology and cell culture purity of **a** primary rhesus astrocytes (*GFAP*, *red*), **b** primary rhesus microglia (*IBA*-*1*, *green*), and **c** human oligodendrocytes (*MBP*, *green*). The nuclei of all cells in **a**–**c** appear *blue* due to staining with the nuclear stain TOPRO3. The two-way ANOVA and Tukey’s multiple comparison test were used to evaluate the statistical significance between means and SEM of triplicate data sets for astrocytes and microglia, and the mean and SD from two assessments of human oligodendrocytes, ***p < 0.001

A mediator predominantly released by microglia in vitro and into the CSF of rhesus macaques with Lyme meningitis is the chemokine CCL2 [16, 20]. Therefore, we evaluated the ability of different concentrations of dexamethasone and meloxicam to reduce CCL2 levels induced by Bb in cultures of primary rhesus astrocytes and microglia, as well as human oligodendrocytes after 48 h of incubation. As expected, dexamethasone (Fig. 2b, e, h) but not meloxicam (Fig. 2c, f, i) significantly reduced CCL2 levels at the drug concentrations tested. Moreover, the anti-inflammatory effect of the dexamethasone formulation was confirmed to be due to its dexamethasone fraction and not due to the carrier substance (2-hydroxypropyl)-β-cyclodextrin (HPC) (Fig. 2b, e, h).

The effect of these two anti-inflammatory drugs was also assessed using a multiplex analysis of cytokines and chemokines that were released from the studied glial cells in the presence of Bb. Table 1 summarizes these results and shows that multiple mediators are significantly affected by dexamethasone but not by meloxicam.

**Table 1:** Cytokines and chemokines are induced by Bb in glial cells of the CNS and significantly reduced by dexamethasone (when used at concentrations of 150 μM; *p* < 0.001), as evaluated in culture supernatants after 48 h. Meloxicam had no effect

Rhesus astrocytes			Rhesus microglia			Human oligodendrocytes		
Mediator pg/mL	Bb	Bb + dex	Mediator pg/mL	Bb	Bb + dex	Mediator pg/mL	Bb	Bb + dex
IL-1β	24	0	IL-1β	64 ± 10	0	IL-6	141 ± 1.2	65 ± 2.7
IL-2	146	21.8 ± 10	IL-2	268 ± 12	54	IL-8	1145 ± 609	97 ± 2.2
IL-6	27175 ± 2567	596 ± 51	IL-6	92562 ± 2979	1319 ± 77	CCL2	5691 ± 410	1898 ± 96
IL-8	113486 ± 882	1636 ± 40	IL-8	493804 ± 3550	3069 ± 309			
IL-10	36	3.72	IL-10	40.45 ± 6.26	17.94			
CCL2	76594 ± 1076	2442 ± 260	CCL2	256397 ± 2581	12415 ± 409			
CCL3	95.43	0	CCL3	2948 ± 176	136 ± 10			
CCL4	25.34	0	CCL4	183.64	0			
VEGF	1220 ± 122	0	VEGF	3153 ± 108	941 ± 28			
GMCSF	674 ± 20	0	GMCSF	3076 ± 127	501 ± 6.14			
			TNF-α	569 ± 45	0			

### Dexamethasone protects FC neurons and oligodendrocytes from Bb-induced apoptosis

**Fig. 3:**
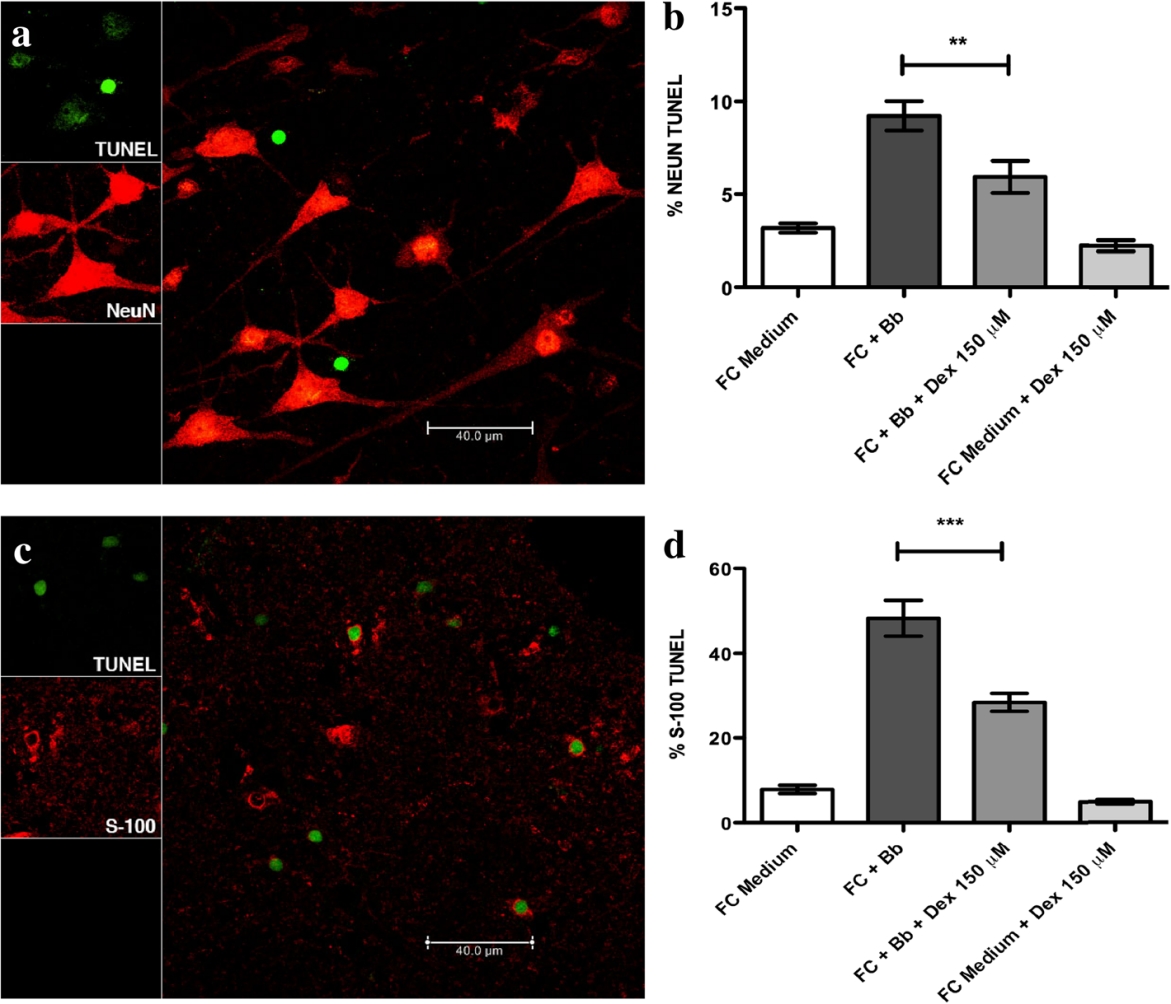
Dexamethasone protects FC neurons (**a, b**) and oligodendrocytes (**c, d**) from Bb-induced apoptosis. Representative images of apoptosis after immunofluorescence staining and visualized by confocal microscopy by the in situ TUNEL assay (*green*) in **a** neurons stained with NeuN (*red*), and **c** oligodendrocytes stained with S-100 (*red*). **b, d** Graphical representations of the effect of dexamethasone on the levels of apoptosis in neurons and oligodendrocytes, respectively, as induced by live Bb. The two-way ANOVA and Tukey’s multiple comparison test were used to evaluate the statistical significance between means and SD of ten data sets (approximately 500 cells) for each condition, **p < 0.01, ***p < 0.001

Live Bb induced enhanced levels of apoptosis, as measured by the TUNEL assay, in both neurons and oligodendrocytes as compared to medium alone. A representative confocal image of apoptosis in the presence of live Bb, as visualized by the in situ TUNEL assay, and a graph of the quantification levels of dexamethasone protection in neurons are shown in Fig. 3a, b and in oligodendrocytes in Fig. 3c, d.

### Meloxicam affects levels of COX-2 induced by Bb in in vitro cultures of rhesus microglia but not of astrocytes

**Fig. 4:**
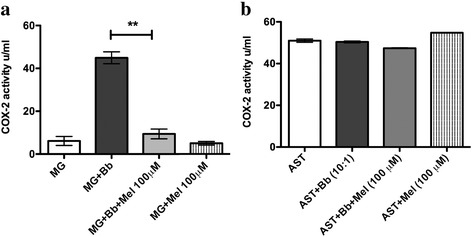
Meloxicam’s effect on COX-2 levels induced by live Bb in rhesus: **a** microglia and **b** astrocytes after 24 h of incubation. The two-way ANOVA and Tukey’s multiple comparison test were used to evaluate the statistical significance between means and SEM of triplicate data sets for microglia and astrocytes, **p < 0.01

COX-2 is known to be involved in both inflammation and apoptosis in brain disorders [21]. As meloxicam is a known COX-2 inhibitor, we evaluated whether it was capable of altering the levels of COX-2 induced by live Bb in cell lysates of rhesus astrocytes and microglia. Results showed that at a concentration within the range used in our assays, this non-steroidal anti-inflammatory drug had an effect in microglia but not in astrocytes (Fig. 4). However, COX-2 appears to be constitutively expressed in astrocytes (Fig. 4b). Thus, meloxicam significantly reduced COX-2 levels as induced by Bb in rhesus microglia (Fig. 4a) but was unable to reduce the levels of pro-inflammatory cytokines and chemokines (Fig. 2c, f, i). These results suggest a cell type differential expression of COX-2 and demonstrate that meloxicam is able to exert its effects in specific cell targets where COX-2 levels are upregulated by Bb.

## Discussion

Once inside the CNS, Bb spirochetes elicit inflammatory mediators from glial and neuronal cells, as well as lymphomonocytic pleocytosis in the CSF [11, 12, 16, 22, 23]. Research models have allowed insights into the pathogenesis of LNB, and although several studies suggest that inflammation is required to trigger apoptosis of neurons and glial cells, a causal relationship between these two phenomena had not been demonstrated. Recently, we addressed the hypothesis that inflammation is a key factor in LNB pathogenesis by using two well-known anti-inflammatory drugs, dexamethasone and meloxicam, in a rhesus macaque model. Results revealed that dexamethasone but not meloxicam treatment effectively suppressed inflammation due to exposure to Bb, and this resulted in inhibition of glial and neuronal damage [11]. To corroborate this finding, our in vitro studies using PNS cells, namely, primary DRG neurons and human Schwann cell cultures also explored the effects of such drugs in the presence of Bb [13]. Dexamethasone and meloxicam demonstrated to have differential anti-inflammatory effects, with dexamethasone being the only one able to significantly reduce Bb-induced immune mediators and apoptosis of PNS cells.

Here, we assessed the ability of dexamethasone and meloxicam to affect inflammation and apoptosis in CNS cells and tissues. To this end, we evaluated the levels of Bb-induced inflammatory mediators in culture supernatants of rhesus FC explants (Fig. 1) and purified primary rhesus astrocytes and microglia and human oligodendrocytes (Fig. 2). We also ascertained the potential of dexamethasone to modulate Bb-induced neuronal and oligodendrocyte apoptosis in rhesus FC explants (Fig. 3). The compiled results from this study help to explain our in vivo findings of significantly reduced inflammatory mediators in the CSF and lack of inflammatory neurodegenerative lesions in the brain and spinal cord of Bb-infected animals that were treated with dexamethasone [11].

The Bb genome encodes, approximately, 150 lipoproteins that play an important role in disease pathogenesis and host immunity [24]. The majority of the pro-inflammatory lipoproteins are outer surface proteins, and their differential expression in various tissues and at different times during infection appear to be critical determinants of disease [17, 25]. An effective host response will contain or clear infections. However, if this response is continually activated, it may lead to lesion development and disease. It is still a matter of debate how spirochetes pass the blood–brain barrier, but hematogenous dissemination appears to be a suitable way [2]. Once they enter the CNS, Bb encounter immune cells such as monocytes, macrophages, or dendritic cells, as well as glial cells such as microglia and astrocytes, all of which produce pro-inflammatory cytokines, e.g., IL-6, IL-8, IL-12, IL-18, and IFNγ, and chemokines such as I-TAC, CCL2, CXCL-11, and CXCL13, as found in the CSF of patients [26–30] and rhesus macaques with LNB [11, 14]. In this study, we have demonstrated, with CNS cells and tissues, that a continuous inflammatory response is detrimental and leads to neural cell death.

Previously, we have shown that meloxicam was not a viable adjunctive therapeutic agent for the treatment of Bb-induced inflammation [11, 13]. Our present ex vivo and in vitro experiments (Fig. 1 and Table 1) confirm this finding. Meloxicam is analgesic and antipyretic; has a high gastric and renal tolerance; a high therapeutic index; and preferentially inhibits COX-2 [31]. To confirm that meloxicam effectively acted as a COX-2 inhibitor at the concentrations used in our study, we evaluated if this drug could dampen the level of this enzyme, as induced by live Bb in cell lysates of primary rhesus astrocytes and microglia. As shown in Fig. 4, meloxicam significantly reduced COX-2 levels from Bb-infected microglia (Fig. 4a), while it was unable to alter the constitutive levels of COX-2 in astrocytes (Fig. 4b). This verifies, on the one hand, that COX-2 is not causally involved in the inflammation by microglia in response to Bb, as indicated by the lack of microglial anti-inflammatory response to meloxicam, and on the other, that astrocytes do not respond to Bb by elevating the expression of COX-2.

Antibiotics will continue to be the first-line therapy for Lyme borreliosis. Steroids have been administered alongside antibiotics, with the literature reporting some beneficial effects [32, 33]. However, opposite results have been obtained as well in cases of facial palsy associated with Lyme neuroborreliosis [34]. Thus far, the findings from our group included in vivo, ex vivo, and in vitro works that used different neuronal and glial cells and suggested a protective role of dexamethasone in LNB. However, the implications of dexamethasone with regard to the treatment of human disease are not clear. What is known is that dexamethasone interferes with pro-inflammatory signal transduction, gene expression, and protein synthesis at various levels [35, 36]. The actions of dexamethasone in bacterially induced TLR-mediated pro-inflammatory signaling involve inhibition of IκBα phosphorylation and degradation as well as NF-κB DNA-binding activity [35]. Moreover, MEK, JNK, and p38, all belonging to the family of MAPKs, are prominent targets of dexamethasone [37–40]. A recent study from our group showed that the TLR2 pathway plays a predominant role in inducing CNS cell inflammation in response to Bb and that the downstream signaling involves the MyD88 and MAPK pathways [41]. Accordingly, it is possible that dexamethasone inhibits Bb-induced TLR2 signaling and the MEK/ERK pathway, reducing inflammation and subsequent apoptosis. Overall, our data suggest that inflammation has a causal role in the pathogenesis of CNS LNB. Further evaluation of its signaling and immunomodulatory mechanisms is still required to ascertain which inhibitors of inflammation may be safely used to mitigate the signs and symptoms of LNB.

## Conclusions

These results indicate that dexamethasone and meloxicam have differential anti-inflammatory effects on Bb-induced inflammation in glial and neuronal cells of the CNS and help explain the in vivo findings of significantly reduced inflammatory mediators in the CSF and lack of inflammatory neurodegenerative lesions in the brain and spinal cord of Bb-infected animals that were treated with dexamethasone but not meloxicam. Signaling cascades altered by dexamethasone could serve as possible therapeutic targets for limiting CNS inflammation and tissue damage in LNB.

## Abbreviations

Bb: *Borrelia burgdorferi*
BSK: Barbour-Stoenner-Kelly
COX: Cyclooxygenase
CSF: Cerebrospinal fluid
ELISA: Enzyme-linked immunosorbent assay
LNB: Lyme neuroborreliosis
MBP: Myelin basic protein
MOI: Multiplicity of infection
MTT: 3-(4,5-dimethylthiazol-2-yl)-2,5-diphenyltetrazolium bromide
PBS: Phosphate-buffered saline
PNS: Peripheral nervous system
TUNEL: Terminal deoxynucleotidyl transferase dUTP nick end labeling
